# The N-terminal Helix Is a Post-assembly Clamp in the Bacterial Outer Membrane Protein PagP

**DOI:** 10.1016/j.jmb.2007.07.072

**Published:** 2007-10-26

**Authors:** Gerard H.M. Huysmans, Sheena E. Radford, David J. Brockwell, Stephen A. Baldwin

**Affiliations:** 1 Astbury Centre for Structural Molecular Biology, University of Leeds, Leeds, LS2 9JT, UK; 2 Institute of Membrane and Systems Biology, University of Leeds, Leeds, LS2 9JT, UK; 3 Institute of Molecular and Cellular Biology, University of Leeds, Leeds, LS2 9JT, UK

**Keywords:** *di*C_12:0_PC, diC_12:0_-phosphatidylcholine, FTIR, Fourier transform infrared spectroscopy, Gdn-HCl, guanidinium chloride, LDAO, lauryldimethylamine-oxide, LUVs, large unilamellar vesicles, PFO, perfluoro-octanoic acid, pNPP, *p*-nitrophenylpalmitate, SUVs, small unilamellar vesicles, membrane protein folding, PagP, outer membrane protein, β-barrel, stability

## Abstract

The *Escherichia coli* outer membrane β-barrel enzyme PagP and its homologues are unique in that the eight-stranded barrel is tilted by about 25° with respect to the membrane normal and is preceded by a 19-residue amphipathic α-helix. To investigate the role of this helix in the folding and stability of PagP, mutants were generated in which the helix was deleted (Δ(1-19)), or in which residues predicted to be involved in helix–barrel interactions were altered (W17A or R59L). The ability of the variants to insert into detergent micelles or liposomes was studied *in vitro* using circular dichroism, fluorescence, Fourier transform infrared spectroscopy, electrophoretic mobility and gain of enzyme activity. The data show that PagP, initially unfolded in 5% (w/v) perfluoro-octanoic acid or 6 M guanidinium chloride, inserts spontaneously and folds quantitatively to an active conformation into detergent micelles of cyclofos-7 or into large vesicles of diC_12:0_-phosphatidylcholine (*di*C_12:0_PC), respectively, the latter in the presence of 7 M urea. Successful refolding of all variants into both micelles and liposomes ruled out an essential role for the helix or helix–barrel interactions in folding and membrane insertion. Measurements of thermal stability indicated that the variants R59L, W17A/R59L and Δ(1-19) were destabilised substantially compared with wild-type PagP. However, in contrast to the other variants, destabilisation of the W17A variant relative to wild-type PagP was much greater in liposomes than in micelles. Analysis of the kinetics of folding and unfolding of all variants in *di*C_12:0_PC liposomes suggested that this destabilisation arises predominantly from an increased dissociation of the refolded variant proteins from the lipid-inserted state. The data support the view that the helix of PagP is not required for folding and assembly, but instead acts as a clamp, stabilising membrane-inserted PagP after folding and docking with the membrane are complete.

## Introduction

Integral membrane proteins are typically categorised into two classes according to the fold of their transmembrane domains which can either be predominantly α-helical in nature or form a closed β-barrel.^1^ Members of the β-barrel class of membrane proteins usually form very stable entities in the membrane, either as monomers (e.g. OmpA),^2^ or as oligomeric assemblies (e.g. OmpF).^3^ It is generally accepted that the extensive hydrogen bond network between the transmembrane strands forms the basis of the extreme stability of these proteins.^4^

*Escherichia coli* PagP and its homologues are β-barrel membrane proteins found in the outer membranes of a number of Gram-negative bacteria, including *Salmonella* and other pathogens.^5^ PagP transfers a palmitate chain from phospholipids to the lipid A moiety of lipopolysaccharides in the outer leaflet of the membrane, reinforcing the hydrocarbon core of the outer leaflet.^6^ Such a reinforced membrane protects the bacterium from host immune defenses by (i) counteracting scavenging of Mg^2+^ by cationic antimicrobial peptides^7,8^ and (ii) attenuation of the ability of lipid A to activate these immune responses.^5^

**Figure 1:**
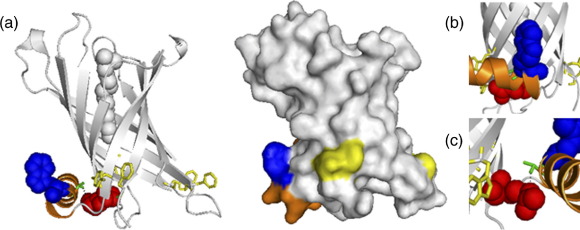
(a) Ribbon diagram (left) and surface model (right) of PagP. An LDAO molecule (grey space fill) was modelled in the hydrophobic substrate pocket of the protein (left). (b) Detail of the position of Trp17 with respect to the barrel face. (c) Detail of Arg59 forming a hydrogen bond with the side-chain of Thr16 in the N-terminal α-helix. In all Figures the N-terminal α-helix is shown in orange, Arg59 in red, Trp17 in blue and Thr16 in green. Residues that complete the periplasmic aromatic girdle (Tyr23, Trp89, Trp93, Phe101, Tyr133, Phe161) are shown in yellow. The images were drawn using PyMol [http://www.pymol.org] (PDB-code, 1THQ)^11^.

The three-dimensional structure of the 161-residue PagP protein has been solved both by solution NMR^9,10^ and by X-ray crystallography.^11^ The 141 C-terminal residues form an eight-stranded transmembrane β-barrel, preceded by a short amphipathic α-helix, 19 residues in length (Figure 1(a)). Both the NMR and X-ray structures suggest that PagP is monomeric, although dimers have been observed after solubilisation in lauryldimethylamine-oxide (LDAO) micelles.^12^ The transmembrane β-barrel of PagP contains a hydrophobic binding cleft for the phospholipid substrate, oriented perpendicular to the membrane plane (Figure 1(a)). The β-barrel of PagP is proposed to be tilted by 25° with respect to the membrane normal,^11,13^ and it has been suggested that this creates a docking space for the amphipathic helix. The position of this helix with respect to the barrel could not be determined unambiguously from the solution NMR structures in detergent micelles,^10^ but it was found to be closely packed against the barrel in the protein crystals.^11^ In the latter structure packing of the helix protects an otherwise membrane-exposed hydrophilic patch at the periplasmic ends of the second and third β-strands (Figure 1(b)). Additionally, the amphipathic helix contributes a tryptophan residue (Trp17), which completes the aromatic girdle on the periplasmic side of the protein (Figure 1(a)). Recently, further evidence for a close helix–barrel interaction has emerged from studies in which solution NMR was used to probe water and oxygen contacts of PagP in dodecylphosphocholine micelles^13^ and from the *in silico* identification of the helix as an in-plane membrane anchor.^14^ Mutants bearing partial helix deletions have been shown to be able to be translocated across the inner membrane and transported to the outer membrane, and to form a fully active protein *in vivo*.^15^ However, the precise role of the amphipathic α-helix in PagP stability and folding has remained elusive.

Here we have analysed the stability, activity and folding kinetics of full-length mature PagP (lacking a signal peptide) and of mutants designed to perturb the helix–barrel interaction *in vitro* (Figure 1). We characterised these mutants after folding in detergent using a previously published protocol^9^ and in an assay in which folding occurs into pre-formed small and large unilamellar vesicles (SUVs and LUVs, respectively). The results demonstrate that the α-helix and the β-barrel are in close contact in micelles of cyclofos-7, consistent with previous hypotheses^11,13^ and by mutagenesis of Arg59 reveal that this interaction makes a major contribution to the stability of PagP. We also show that the helix is entirely superfluous for membrane insertion into lipid and that Trp17 stabilises PagP in the membrane bound form. By combining these results with kinetic data we propose that in lipid membranes the helix acts as a clamp, locking the protein in the native, active conformation once folding and insertion are complete.

## Results

### Wild-type PagP folds in detergent micelles and in pre-formed liposomes

**Figure 2:**
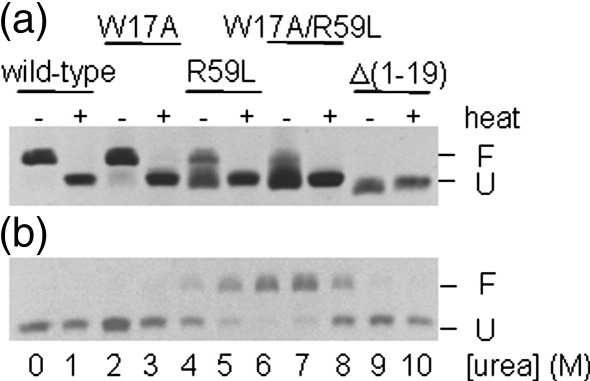
Electrophoretic analysis of PagP refolding. (a) Insertion and folding of different PagP variants into cyclofos-7 micelles. The application of heat to unfold the protein is indicated above the gel. (b) Insertion and folding of wild-type PagP into *di*C_12:0_PC LUVs at different urea concentrations with a lipid-to-protein ratio of 800:1 in 50 mM sodium phosphate buffer (pH 8) at 25 °C. F, folded state; U, unfolded state.

Successful refolding to a functional state by diluting denatured protein into detergent micelles^16–21^ or lipid bilayers^22–28^ has been reported for several bacterial outer membrane β-barrel proteins, as well as the mitochondrial outer membrane protein VDAC.^29^ In many (but not all) such cases, the electrophoretic mobility of the folded proteins on SDS–polyacrylamide gel electrophoresis differs from that of the fully denatured forms if the samples are not boiled prior to electrophoresis (cold SDS–PAGE).^30^ We exploited this phenomenon to provide a simple assay for refolding of PagP in detergent and to investigate the conditions necessary for successful refolding of the protein into pre-formed liposomes. Using this assay, we showed that PagP, initially denatured in perfluoro-octanoic acid (PFO), could be refolded successfully into micelles of cyclofos-7 following a published protocol:^9^ the folded protein migrated with an apparent molecular mass of 21 kDa, which was decreased to 18 kDa when denatured by heat (Figure 2(a)). Although the folded state of β-barrel outer membrane proteins usually migrate faster than the unfolded conformation, inverse shifts for some proteins, including PagP, have been reported.^10,31^ In agreement with the reports of others, PagP refolded from an SDS-denatured state into β-d-octylglucoside showed inverted migration shifts similar to those described above (data not shown). To confirm that the more slowly migrating band on gels of PagP refolded into cyclofos-7 represents the correctly folded protein, the enzymatic activity of the refolded protein towards the substrate analogue *p*-nitrophenylpalmitate (*p*NPP) was measured. In previous studies, the catalytic activity of PagP has been determined by the transfer of palmitoyl chains from various ^32^P-labelled phospholipid substrates to lipopolysaccharides^12^ or by monitoring the cleavage of doubly ^13^C-labelled substrates using NMR.^9^ Here, we developed a new assay in which the release of the *p*-nitrophenol group from *p*NPP was monitored directly at 410 nm in a buffer containing Triton X-100 (see Methods). Addition of Triton X-100 to the assay buffer is necessary to dissolve released palmitate; however, whether Triton X-100 acts as a substrate in a transferase reaction or solubilises palmitate after *p*NPP hydrolysis is currently under investigation. The resulting data demonstrated that PagP refolded into cyclofos-7 attained a functionally active conformation able to hydrolyse *p*NPP and release the coloured product *p*-nitrophenol, whilst heat-denatured PagP (which has a higher electrophoretic mobility) is inactive (see Methods and Table 1). Consistent with its enzyme activity, PagP refolded in this manner gave rise to a well dispersed ^1^H-^15^N-TROSY HSQC spectrum consistent with previously published NMR spectra of the native protein^9,10^ (data not shown).

Some integral β-barrel membrane proteins have been shown to refold spontaneously from a chaotrope-denatured state into pre-formed lipid bilayers.^24,27–29,32,33^ However, dilution of PagP, denatured in 6 M guanidinium chloride (Gdn-HCl), into liposomes consisting of 100% diC_12:0_-phosphatidylcholine (*di*C_12:0_PC) did not lead to a stably folded protein as determined by cold SDS–PAGE (Figure 2(b), lane 1). We chose *di*C_12:0_PC liposomes for refolding experiments because folding and insertion into both SUVs and LUVs with short lipid chain lengths has been reported to produce better yields for other β-barrel proteins.^26,34^ Moreover, the enzymatic activity of PagP towards the shorter hydrocarbon chains of these phospholipids was expected to be minimal.^12^ Building on the observation that the folding and membrane insertion of OmpA is highly dependent on pH^33,35^ and temperature,^25,36^ we attempted the insertion and folding of PagP into *di*C_12:0_PC liposomes through variation of these parameters (pH 7–10; 4–37 °C), but without success (data not shown). In all such conditions PagP precipitated immediately following dilution into the liposome mixture, suggesting a competition between insertion and aggregation. To disfavour aggregation and hence to maintain the protein in an insertion-competent state, we next analysed the folding and insertion of PagP in *di*C_12:0_PC liposomes in the presence of different concentrations of urea. At concentrations of ca 4 M–8 M urea a migration shift in electrophoretic mobility indicative of folding and membrane-insertion was evident both in SUVs made by sonication (data not shown) and in LUVs prepared by extrusion (Figure 2(b)). In the presence of 6–7 M urea 85–90% of PagP migrated as the putatively folded form, whilst increasing the urea concentration further to 10 M led to the complete retention of the apparent unfolded state.

**Figure 3:**
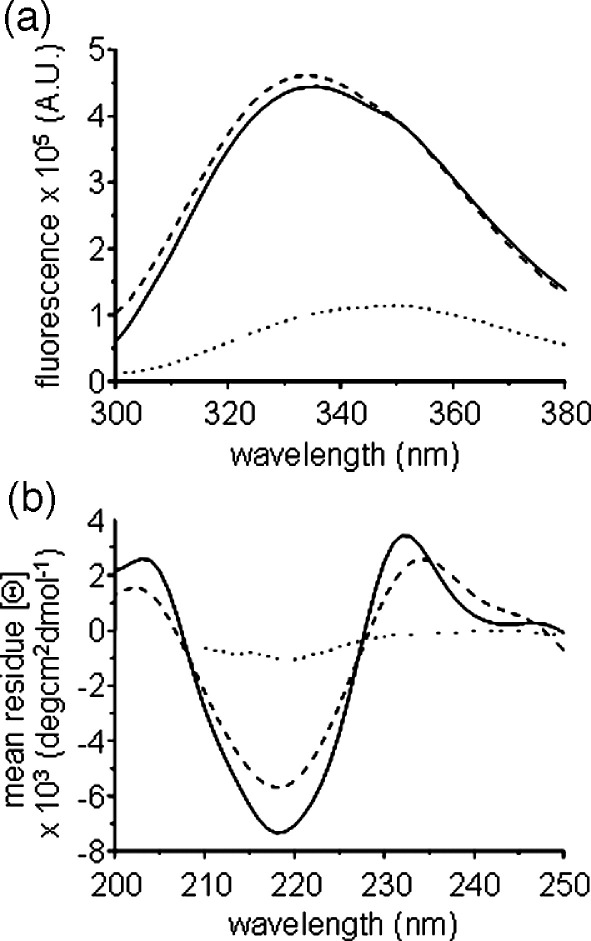
(a) Fluorescence emission spectra of 0.5 μM wild-type PagP and (b) circular dichroism spectra of 5 μM wild-type PagP. (⋯) Unfolded in 8 M urea; (–) refolded in cyclofos-7 and (–––) refolded in *di*C_12:0_PC LUVs. Background spectra without PagP were subtracted. All spectra were recorded at 25 °C in 50 mM sodium phosphate buffer (pH 8).

To compare the structure of PagP refolded into cyclofos-7 with that of protein folded and inserted into *di*C_12:0_PC LUVs, the conformational properties of the protein were examined using tryptophan fluorescence and far UV circular dichroism (CD). For these experiments reconstituted PagP was first separated from non-reconstituted protein by flotation of the liposomes on a sucrose gradient (see Methods). PagP contains 12 tryptophan residues, eight in the transmembrane region of the barrel, two in the extracellular loops and two in the N-terminal region that contains the amphipathic helix.^10^ Tryptophan residues in the transmembrane domains of outer membrane β-barrels typically form aromatic girdles around the protein, located in the interfacial regions of the lipid bilayer.^37–40^ Burial of eight tryptophan residues upon refolding of PagP and its insertion into detergent micelles or the lipid bilayer would be expected to cause a change in intensity and a blue shift of the fluorescence emission maximum. Consistent with this expectation, fluorescence emission spectra of PagP refolded into cyclofos-7 micelles or *di*C_12:0_PC LUVs were characterised by a substantial increase in fluorescence intensity and a shift in emission maximum from 350 nm for the unfolded protein in 8 M urea to approximately 335 nm for refolded PagP (Figure 3(a)). Retention of a small shoulder at 350 nm in the spectrum of the refolded protein may reflect the presence of the two solvent-exposed tryptophan residues on the extracellular loops of PagP.

The far UV CD spectra of cyclofos-7-refolded and liposome-reconstituted PagP are similar to reported spectra of PagP in LDAO (small spectral differences at very low wavelengths in some samples presumably resulting from light scattering effects).^41^ Both spectra of the refolded protein exhibit a minimum at 218 nm, consistent with the protein folding to a predominantly β-sheet conformation, which is absent in the spectrum of unfolded PagP in 8 M urea (Figure 3(b)). A band with a positive molar ellipticity around 232 nm is also observed in the spectrum of both refolded proteins. This band has been attributed mainly to a Cotton effect between Tyr26 and Trp66,^41^ two residues which are distant in sequence space, but which pack closely in native PagP. The presence of this band is thus highly characteristic of native PagP. Together with the observed changes in electrophoretic mobility and tryptophan fluorescence emission spectra, these data demonstrate that PagP is able to refold into its native conformation both in cyclofos-7 and in *di*C_12:0_PC LUVs.

### Kinetics of PagP refolding in pre-formed liposomes

**Figure 4:**
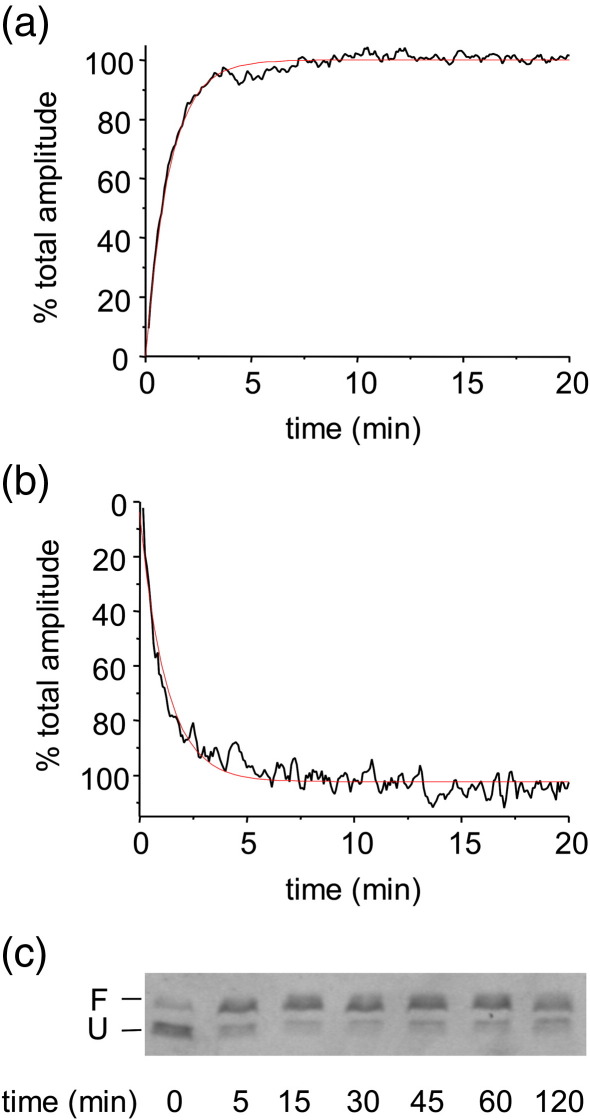
Folding kinetics of wild-type PagP initially denatured in 6 M Gdn-HCl into *di*C_12:0_PC LUVs monitored by (a) CD at 232 nm; (b) CD at 218 nm and (c) SDS–PAGE. All experiments were performed at 25 °C in the presence of 7 M urea at pH 8 and a lipid-to-protein ratio of 800:1. The data in (a) and (b) are fitted to a single exponential (red line) The final protein concentration was 5 μM.

Insights into the folding kinetics of PagP were next obtained by monitoring changes in the electrophoretic mobility and far UV CD signal of the protein (initially denatured in 6 M Gdn-HCl) when diluted into buffer containing *di*C_12:0_PC LUVs and 7 M urea (Figure 4). The time courses of secondary and tertiary structure formation were examined using far UV CD. Thus, by monitoring the increase in ellipticity at 232 nm (Figure 4(a)) the formation of the native core characterised by the Tyr26–Trp66 phenol-indole ring interaction could be measured, whilst the formation of secondary structure was followed using the β-sheet CD signal at 218 nm (Figure 4(b)). These time courses could be fitted to single exponentials with rate constants of 0.88(±0.01) min^−1^ and 0.92(±0.03) min^−1^, respectively. Consistent with this, gel shift assays showed the quantitative formation of stable structure, resistant to complete SDS–denaturation, within 5 min of initiating refolding (Figure 4(c)), whilst activity assays using *p*NPP were used to show acquisition of enzyme activity. Although the latter assays do not allow a quantitative comparison with the PagP activity in cyclofos-7 micelles, since the precise substrate concentration is not well defined in the presence of liposomes, they qualitatively show that enzyme activity is present within 2 min after initiation of refolding in *di*C_12:0_PC liposomes (data not shown). Together the data demonstrate that the folding and membrane insertion of PagP into a catalytically active state in *di*C_12:0_PC liposomes are complete within ∼5 min of initiating refolding.

### Role of the amphipathic helix in PagP folding and stability

A characteristic feature of PagP and its homologues is the presence of an N-terminal amphipathic α-helix. Based on the crystal structure, this helix is predicted to interact both with the β-barrel and with the inner leaflet of the outer membrane bilayer, although these interactions were not detected in the NMR structures of the protein in detergent micelles.^10,11^ Residue Trp17, which is absolutely conserved in PagP and 12 homologues, has been proposed to contribute to a girdle of aromatic residues stabilising the protein *via* interactions with the interfacial region of the bilayer at the periplasmic surface of the membrane^11^ (Figure 1(a) and (b)). Interestingly, mutation of Trp17 to Ala has no effect on protein activity.^15^ The second residue that may form a key interaction between the helix and the β-barrel, Arg59 in strand 2 of the β-barrel, is also highly conserved (in 10 of the 13 sequences of PagP and its homologues). This residue is predicted to form a hydrogen bond from its side-chain to the side-chain hydroxyl of Thr16 in the N-terminal helix (conserved in 8 of the 13 sequences), and to provide packing interactions with the helix (Figure 1(a) and (c)). To assess the significance of these interactions in PagP folding and stability, we examined the effect of removing them by creating the variants W17A and R59L, as well as the double mutant protein, W17A/R59L. Additionally, the entire helix was deleted in the variant Δ(1-19). The ability of these proteins to fold to an active state in cyclofos-7 was then assessed by examining their ability to hydrolyse *p*NPP. In all cases the activities of the mutant proteins were similar to that of the wild-type PagP (Table 1), mirroring the findings of Jia *et al.* that the W17A variant of PagP, and PagP variants lacking various parts of the helix, are able to fold to a catalytically active conformation *in vivo*.^15^ In addition, analysis of the secondary structural content of the refolded PagP proteins in cyclofos-7 micelles using Fourier transform infrared spectroscopy (FTIR) revealed a β-sheet content similar to that of wild-type PagP for the variants created, whilst Δ(1-19) showed the expected decrease in helical content, with a concomitant increase in the percentage of β-sheet structure (Table 2). Together the data show that the mutant proteins created are able to fold in cyclofos-7 to a native, active conformation, demonstrating that the helix is fully dispensable for *in vitro* folding in detergent and for the formation of catalytically active PagP.

### The N-terminal α-helix stabilises PagP in detergent

**Figure 5:**
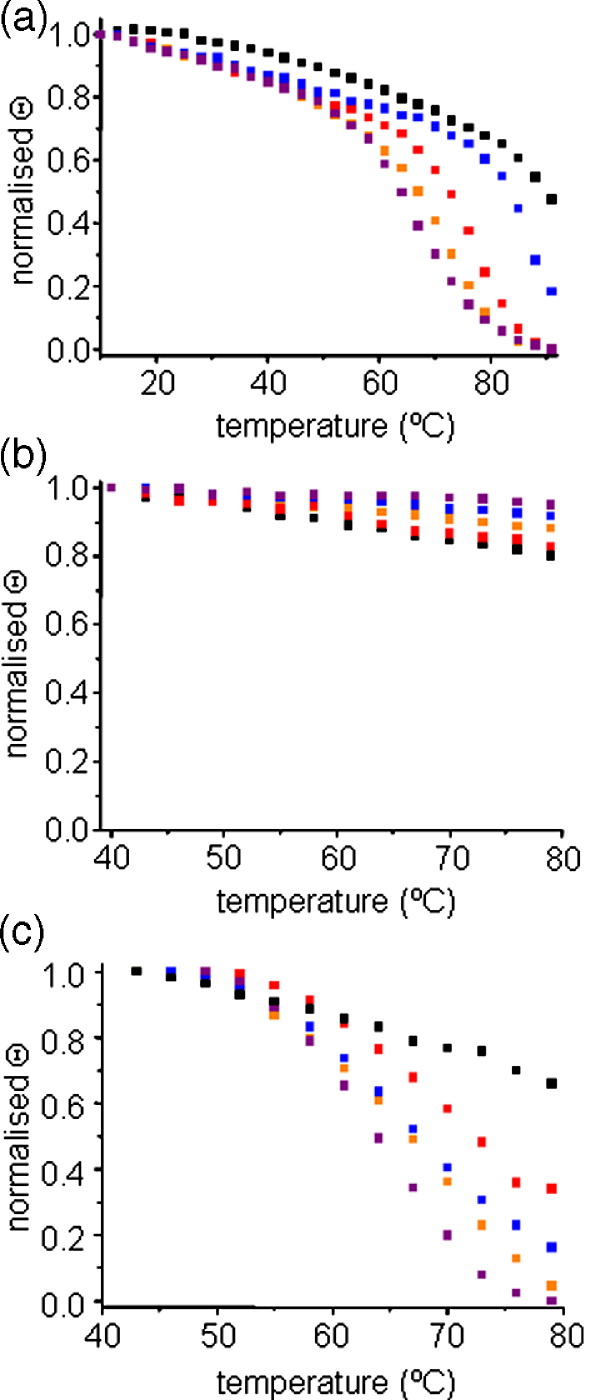
Thermal denaturation curves determined by changes in molar ellipticity at 232 nm of PagP refolded in (a) cyclofos-7, (b) *di*C_12:0_PC LUVs and (c) *di*C_12:0_PC LUVs in the presence of 7 M urea. Wild-type PagP is in black, W17A in blue, R59L in red, W17A/R59L in purple and Δ(1-19) PagP in orange. The temperature was increased in steps of 3 °C. Data points were normalised to the signals for folded and unfolded protein (see Methods).

Despite the lack of effect of mutating residues involved in helix–barrel interactions on catalytic activity, the results of gel shift analysis of non-heat-denatured samples suggested that perturbation of the helix-barrel interactions decreases PagP resistance to SDS denaturation (Figure 2(a)). These experiments suggested that whilst the stability of W17A is very similar to that of the wild-type protein, the variants R59L, W17A/R59L and Δ(1-19) are significantly destabilised, since these variants lacked, or partially lacked, an SDS-resistant structure. Variant Δ(1-19), which did not yield a stable gel shift, was confirmed to have a native-like fold in cyclofos-7 using ^1^H-^15^N-TROSY HSQC NMR experiments (data not shown). Further analysis of the stability of the mutant proteins in cyclofos-7 micelles was obtained using thermal unfolding monitored at 232 nm using far UV CD (Figure 5(a)). The extreme stability of wild-type PagP in cyclofos-7 is exemplified by the observation that a temperature of 91 °C is not sufficient to drive unfolding to completion. Complete, though irreversible, unfolding of wild-type PagP could be achieved, however, at 88 °C in cyclofos-7 after the addition of 4% SDS (data not shown).

The rank order of thermal stability of the PagP variants measured by far UV-CD is very similar to that obtained using gel shift assays. Thus, the stability of W17A was reduced only slightly compared with wild-type PagP, indicating only a minor contribution of this residue to the stability of the protein in cyclofos-7 (Figure 5(a)). However, the R59L mutation decreased PagP stability to a greater extent. Combining both the W17A and R59L mutations yielded an additive effect on protein stability, such that the resulting double mutant protein had a stability close to that of Δ(1-19): both these variants of PagP unfolded completely, though irreversibly, within the accessible temperature range (Figure 5(a)). These findings suggest that these two residues provide the vast majority of stabilising interactions between the helix and the β-barrel. The data therefore yield an order of thermal stability of wild-type > W17A > R59L > Δ(1-19) ∼ W17A/R59L.

### The N-terminal helix stabilises PagP in pre-formed liposomes

To establish whether the differing stabilities of the PagP variants were also mirrored in their membrane-inserted counterparts, wild-type PagP and the four variants were next refolded into *di*C_12:0_PC LUVs in the presence of 7 M urea. Enzyme activity towards *p*NPP was obtained for each PagP variant following such refolding (data not shown). To compare the thermal stability of all PagP proteins with the results obtained in cyclofos-7, urea was removed after reconstitution of the protein. Retention of folded and inserted PagP in *di*C_12:0_PC LUVs was ascertained by tryptophan fluorescence and perturbation of this fluorescence using KI in quenching experiments, performed after removal of protein aggregates on a sucrose gradient. All variants had Stern–Volmer constants equal to wild-type PagP, which were smaller than that measured for *N*-acetyl tryptophanamide, suggesting that all PagP proteins were folded and inserted into the membrane (Table 1).

By contrast with the thermal unfolding data obtained in detergent, wild-type PagP and its variants were much more stable in LUVs of *di*C_12:0_PC: none of the variant proteins nor wild-type PagP unfolded before the maximum accessible temperature (80 °C; Figure 5(b)). Thermal unfolding of these proteins in *di*C_12:0_PC LUVs was therefore also measured in the presence of 7 M urea. Under these conditions wild-type PagP retains significant native structure and remains membrane-associated even at 79 °C. By contrast, all of the variants created showed substantial destabilisation relative to the wild-type protein. Most dramatically, whilst the variant W17A showed similar thermal stability to wild-type PagP in cyclofos-7 (Figure 5(a)), this variant was substantially destabilised in *di*C_12:0_PC LUVs in 7 M urea, showing a stability similar to Δ(1-19) (Figure 5(c)). Indeed, both the W17A and Δ(1-19) variants unfolded with an apparent midpoint of ∼68 °C. The variants R59L and W17A/R59L were also significantly destabilised relative to wild-type PagP in both cyclofos-7 and in *di*C_12:0_PC LUVs.

### Kinetics of insertion/folding and unfolding of PagP are affected by the N-terminal α-helix

**Figure 6:**
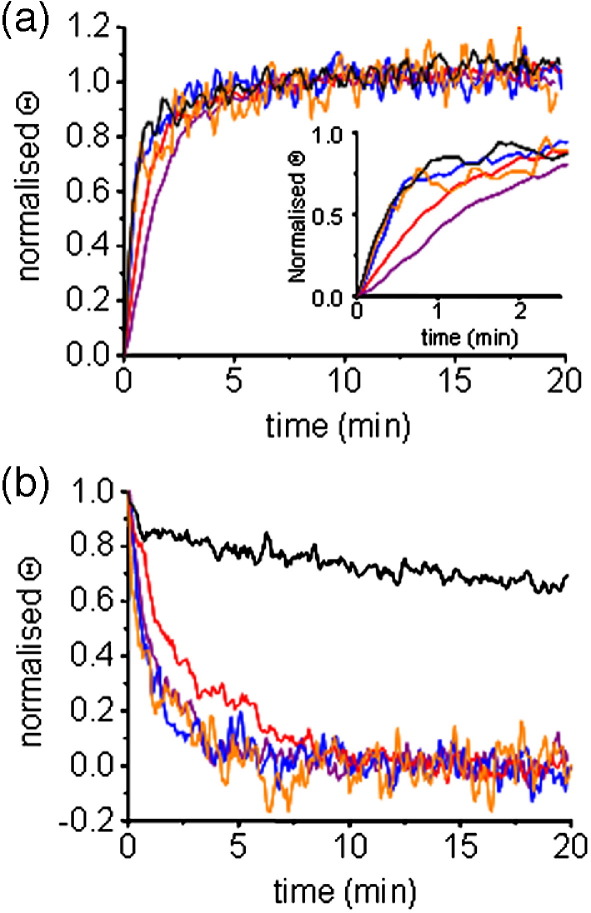
(a) Folding and (b) unfolding kinetics of PagP followed by circular dichroism at 232 nm of wild-type (black), W17A (blue), R59L (red), W17A/R59L (purple) and Δ(1-19) PagP (orange). CD experiments were conducted using 5 μM PagP at 37 °C in the presence of 7 M urea for folding experiments and 10 M urea for unfolding experiments with a lipid-to-protein ratio of 800:1 in *di*C_12:0_PC LUVs. The inset in (a) shows an expansion of the folding data over the first 2. 5 min. Traces were normalised to the signals for folded and unfolded protein to enable comparison of kinetics (see Methods).

The differences in stability of wild-type PagP and its variants in *di*C_12:0_PC LUVs could have arisen either from a decreased rate of folding and insertion of the variants into liposomes or from an increase in their rate of unfolding and membrane dissociation, or from both. To determine the origin of the differences in stability of the variants created, the rate of folding and membrane insertion of each protein, denatured initially with 6 M Gdn-HCl, into *di*C_12:0_PC LUVs in the presence of 7 M urea was measured for all variants using CD at 37 °C. We performed these experiments at 37 °C rather than 25 °C to increase the refolding yield for the variants R59L and W17A/R59L. Interestingly, whilst wild-type PagP and variants W17A and Δ(1-19) differed markedly in their thermal stability, no differences in the kinetics of their folding and insertion into LUVs in 7 M urea were observed, the apparent rates being 1.60(±0.08), 1.52(±0.09) and 1.38(±0.13) min^−1^, respectively (Figure 6(a)). By contrast, the variants R59L and W17A/R59L folded and inserted into these LUVs more slowly than the wild-type protein (apparent rates of 0.86(±0.02) and 0.59(±0.01) min^−1^, respectively). Overall, however, the changes in the rate constants were small relative to the very large difference in thermal stability of the membrane-inserted states (Figure 5(c)).

To complement these experiments the effects of the mutations on the unfolding and dissociation of PagP from membranes were also examined using an assay in which wild-type or mutant PagP was first inserted into *di*C_12:0_PC LUVs in 7 M urea. The urea concentration was then increased to 10 M in order to displace the equilibrium towards the unfolded state and the rate of unfolding was monitored using CD (see Methods). Strikingly, all mutants lacking Trp17 (i.e. W17A, W17A/R59L and Δ(1-19)) unfolded much more rapidly than wild-type PagP, with apparent unfolding rates of 0.76(±0.02), 0.71(±0.02), 0.65(±0.03) and 0.013(±0.001) min^−1^, respectively, whilst the rate of unfolding of R59L was 0.38(±0.02) min^−1^ (Figure 6(b)). Thus, whilst the variants W17A, W17A/R59L and Δ(1-19) had completely unfolded within 5 min, wild-type PagP took over two hours to completely unfold from the membrane. Combined with the equilibrium studies shown in Figure 5(c), therefore, the unfolding kinetics demonstrate a key role for the side-chain of Trp17 for maintaining PagP as a stably folded entity in lipid bilayers, by disfavouring dissociation of the lipid-inserted and folded state. By contrast, Trp17 appears to play little role in PagP stability in detergent micelles (Figure 5(a)).

## Discussion

### Folding of PagP into detergent micelles and liposomes

To date, detailed studies of the mechanism of folding and assembly of β-barrel membrane proteins *in vitro* have mainly focussed on the outer membrane β-barrel proteins, OmpA^24,25,27,32–34,36,42^ and, more recently, FomA^26,42^ and the eukaryotic outer membrane protein VDAC.^29^ Here we report on the thermodynamic and kinetic analysis of the (un)folding of wild-type PagP, an eight-stranded transmembrane β-barrel with an N-terminal amphiphatic α-helix, and of variant proteins, to address the role of the N-terminal α-helix in folding and stability of this protein. In order to measure the thermal and kinetic stability of PagP and variants, it was necessary to fold the protein to a functional form. Previous studies had shown that wild-type PagP could be folded successfully in the detergent cyclofos-7.^9^ Here we reproduced these data showing, in addition, that the protein can be refolded both into SUVs and LUVs of *di*C_12:0_PC. However, by contrast with OmpA and VDAC, which appear to collapse into a water soluble partly folded conformation prior to membrane insertion,^25,29^ PagP folding into vesicles required the presence of significant concentrations of urea: refolding yields close to 90% were obtained in the presence of urea concentrations of 6–7 M. We suggest that this concentration of denaturant is necessary to maintain PagP in a soluble and membrane-insertion-competent state whilst disfavouring protein aggregation, thus allowing successful protein folding and insertion into lipid bilayers. Periplasmic chaperones such as Skp and SurA may play a similar role *in vivo*.^43^

Using a combination of CD, FTIR, fluorescence, Stern–Volmer analysis, SDS–gel migration assays and activity assays, we show here that PagP refolds in cyclofos-7 or into lipid vesicles to a conformation with native-like secondary and tertiary structure and which is catalytically active. Importantly, in the far UV CD spectra, a Cotton effect between Tyr26 and Trp66 gives rise to a maximum in the spectrum around 232 nm.^41^ This band provides an extremely sensitive assay for the formation of the native enzyme. A comparison of the kinetics of formation of this band with those of secondary structure formation measured by CD at 218 nm showed that the rate of formation of secondary structure coincides with the formation of the native fold, consistent with previous reports for OmpA^34,44^ and suggesting that under the conditions utilised the folding of the β-barrel of PagP is cooperative.

### Role of a post-assembly clamp

PagP and its homologues are unusual amongst β-barrel membrane proteins in that the transmembrane β-barrel is extended with an amphipathic α-helix on the periplasmic side of the membrane. Most known structures of outer membrane proteins contain a β-barrel transmembrane domain with short periplasmic turns and longer extracellular loops.^45^ Some transmembrane β-barrels are extended with large soluble domains in the periplasm, such as is the case for OmpA,^46^ or with plug domains that fold back into the β-barrel, such as is found for the TonB-dependent receptors.^47^ The *E. coli* AIDA autotransporter is extended with a short β-sheet domain (the β_1_-domain) on the extracellular side of the protein.^48^ For OmpA and FhuA their periplasmic domains are dispensable for folding *in vivo*.^49,50^ By contrast, the β_1_-domain of AIDA is not required for folding *in vivo*, but it is absolutely necessary for folding *in vitro* in the absence of a solid support.^20^ Here we have shown that deletion of the region containing the N-terminal helix of PagP does not impair the ability of this protein to refold into detergent micelles of cyclofos-7 or into pre-formed vesicles of *di*C_12:0_PC, consistent with previous studies showing that transmembrane β-barrels can fold efficiently *in vitro*.^18,24,28,29,33,51,52^ However, by mutation of conserved residues that stabilise the interaction between the helix and barrel, together with analysis of the thermal stability of the resulting variants, we have demonstrated that the PagP helix contributes significantly to the stability of this β-barrel both in detergent and in LUVs, in the latter case predominantly by decreasing the rate of unfolding and dissociation of the membrane-inserted native state. Thus, although the β-barrel forms an independent folding unit, the N-terminal helix plays a central role in modulating the stability of the native protein. Most importantly, we have shown that the effect of mutating different residues involved in the helix–barrel interaction on protein stability is dependent upon whether PagP is assembled into detergent micelles or into *di*C_12:0_PC LUVs. We show that the side-chain of Arg59 situated in the β-barrel provides most of the stabilising interactions in cyclofos-7: indeed, the stability of the R59L variant resembles that of Δ(1-19), in which the entire helix has been deleted. Mutation of this residue also causes significant destabilisation in *di*C_12:0_PC LUVs. By contrast, mutation of the absolutely conserved Trp17 to Ala produced only minor destabilisation in detergent, but substantial destabilisation in LUVs in 7 M urea, demonstrating that the presence of the indole ring of Trp17 is required to maintain PagP in a stably folded conformation in the lipid bilayer under these conditions. Consistent with this, all variants that lacked Trp17 (W17A, W17A/R59L and Δ(1-19)) were able to fold to an active conformation, but resulted in less stable proteins in *di*C_12:0_PC LUVs that unfold 50-fold more rapidly than wild-type PagP. The data suggest, therefore, that Trp17 plays a crucial role in modulating PagP stability in the bilayer, possibly because the aromatic ring of Trp17 is required to complete the periplasmic aromatic girdle, clamping the β-barrel in the membrane-inserted state after folding of the barrel itself is complete. Rather than being superfluous for folding and assembly of PagP, the N-terminal helix of PagP could play a role in maintaining the integrity of the protein within the lipid bilayer by acting as a post-assembly clamp. Our observations not only provide the first insights into how this family of membrane β-barrel proteins fold, but they also pave the way for more detailed investigations into the mechanisms of PagP folding and membrane insertion, building on our ability to fold and assemble PagP and its variants quantitatively into *di*C_12:0_PC LUVs.

## Methods

### Mutagenesis and protein purification

Site-directed mutants were introduced into plasmid pETCrcAHΔS^10^ using the QuikChange method (Stratagene). Deletion of the N-terminal α-helix was achieved by using inverse PCR to incorporate a unique BamHI restriction site. The subsequent digestion of the PCR product with BamHI and overnight ligation using T4 ligase yielded the construct designated pETCrcAHΔSΔ(1-19). The presence of the desired mutations and the absence of unwanted additional mutations were verified by DNA sequencing. PagP was expressed in *E. coli* strain Rosetta 2 (Novagen) and grown in LB medium (or minimal medium for production of ^15^N-labelled protein). The produced protein was purified from inclusion bodies under denaturing conditions as described.^10^ Typically 50 mg of purified protein was obtained per litre of culture and stored at −20 °C either as a pellet precipitated from the denaturing buffer by dialysis against distilled water or as a solution in 6 M Gdn-HCl, with a typical protein concentration of 0.5 mM.

### Refolding of PagP in cyclofos-7 and SDS–polyacrylamide gel electrophoresis

PagP was refolded in cyclofos-7 (Anatrace, Maumee, OH) using the method described by Hwang *et al*.^9^ Briefly, precipitated PagP was solubilised in 5% (w/v) PFO after which 50 mg cyclofos-7 per 10 mg precipitate was added and dialysed for three days against 50 mM sodium phosphate buffer (pH 6). Successful refolding in these and other experiments was assessed by SDS–polyacrylamide gel electrophoresis,^53^ but without heat-denaturation of the samples (cold SDS–PAGE). To this end aliquots of the reaction mixture were mixed with an equal volume of 100 mM Tris–HCl (pH 6.8), containing 4% (w/v) SDS, 20% (v/v) glycerol and 0.01% (w/v) bromophenol blue and were analysed 10 min later on 15% (w/v) acrylamide gels (37.5:1, acrylamide/bis-acrylamide). Heat-denatured control samples were boiled for at least 10 min prior to analysis. Incubation for longer times both with and without heat resulted in a similar pattern of bands demonstrating that equilibrium had been reached in each case.

### Preparation of liposomes

*di*C_12:0_PC (Avanti, Alabaster, AL, USA) dissolved in a 1:1(v/v) chloroform/methanol mixture was dried on the bottom of a test tube under a gentle stream of nitrogen gas and in a desiccator under high vacuum. The resulting thin lipid films were hydrated to give a 20 mM lipid solution in 50 mM sodium phosphate buffer (pH 8) and briefly vortexed. LUVs were formed by extruding the lipid dispersions 11 times through 100 nm pore-size polycarbonate membranes (Nucleopore, Whatman, Clifton, NJ) using a mini-extruder (Avanti, Alabaster, AL, USA). SUVs were formed by sonication for 45 min, 50% duty cycle at setting 6 (W-225R, Ultrasonics Inc.). Titanium particles were removed by centrifugation (1 min, 13,000***g***).

### Refolding of PagP in pre-formed liposomes

Conditions for the folding of purified and precipitated PagP in *di*C_12:0_PC LUVs were investigated at room temperature by mixing 5 μM denatured PagP in 6 M Gdn-HCl with lipid vesicles in a molar lipid-to-protein ratio of 800:1 in 50 mM sodium phosphate buffer (pH 8) in the presence of 0–10 M urea, typically diluting the Gdn-HCl-containing solution approximately 100-fold. Refolding was assessed by gel-shift analysis as described above. Liposome integrity in the presence of high concentrations of urea was demonstrated using dynamic light scattering.

### Thermal denaturation of PagP in cyclofos-7 and *di*C_12:0_PC liposomes

Refolded PagP in cyclofos-7 was diluted to a final concentration of 5 μM in 50 mM sodium phosphate buffer (pH 8) containing 1% (w/v) cyclofos-7. CD spectra were taken on a Jasco 715 spectropolarimeter between 200 nm and 250 nm using a cell with 1 mm path length, a scan speed of 50 nm min^−1^ and a bandwidth of 1 nm. The temperature was regulated to 25 °C using a Jasco PTC-351S peltier system. For cyclofos-7 refolded PagP, thermal unfolding was monitored by the change in ellipticity at 232 nm resulting from increasing the temperature in 3 °C steps between 10 °C and 91 °C. Highly thermostable wild-type and mutant proteins were also measured by the addition of 2–4% SDS to enable unfolding between 10 °C and 91 °C. Thermal denaturation of liposome-reconstituted PagP was measured between 40 °C and 80 °C (the highest temperature the liposomes were ascertained to be intact as measured by light scattering) in 3 °C steps. For thermal unfolding experiments without urea, the liposomes were pelleted by ultracentrifugation (100,000***g*** for 1 h) prior to resuspension in 50 mM sodium-phosphate buffer (pH 8). Data points were normalised between the signal of folded PagP at 10 °C and unfolded PagP at 91 °C for cyclofos-7 refolded protein according to:

$$sig_{T}(normalised)=\frac{\left(sig_{T}-sig_{91}\right)}{\left(sig_{10}-sig_{91}\right)}$$

where *sig*_*T*_ is the intensity at *T* °C, *sig*_10_ and *sig*_91_ at 10 °C and 91 °C, respectively. For refolded protein in *di*C_12:0_PC LUVs the signal was normalised relative to the signal of folded PagP at 40 °C and unfolded PagP at 79 °C. For mutants that did not unfold completely at the highest accessible temperature the signal of the unfolded state in 2% SDS or 9 M urea was used as the reference signal for unfolded PagP in cyclofos-7 and *di*C_12:0_PC LUV, respectively.

### Fluorescence spectroscopy

Fluorescence emission spectra of 0.5 μM PagP were obtained between 300 nm and 380 nm at 25 °C and a slit width of 2 mm using an excitation wavelength of 280 nm using a Photon Technology International (PTI) fluorimeter (Ford, UK) and a cuvette of 10 mm path length. Similar spectra were obtained using an excitation wavelength of 295 nm. The protein concentration was determined according to Gill & von Hippel.^54^

### Quenching of tryptophan fluorescence

Overnight folding and insertion of PagP into *di*C_12:0_PC LUVs at room temperature was performed as described above in 50 mM sodium phosphate buffer (pH 8) containing 7 M urea. Liposomes were pelleted at 100,000***g*** for 1 h at 4 °C and subsequently separated from non-reconstituted protein aggregates by floating the liposomes on a discontinuous sucrose gradient at 100,000***g*** for 1 h at 4 °C. Pelleted liposomes were then mixed with 500 μl of 40% (w/v) sucrose and overlaid successively with 2.5 ml of 20% sucrose and 300 μl of 0% sucrose. All solutions were made in 50 mM sodium phosphate buffer (pH 8).

Quenching of tryptophan fluorescence was carried out at 25 °C in 50 mM sodium phosphate buffer (pH 8) by increasing concentrations of KI (0–500 mM). Sodium thiosulfate (0.1 M) was added to the iodide solution to prevent I_3_^−^ formation. The fluorescence emission spectrum of the protein in the absence of iodide (*F*_0_) was measured, after which the fluorescence was quenched by progressive addition of small aliquots from the iodide stock solution. Consecutive spectra (*F*) were taken and analysed according to the Stern–Volmer equation:^55^

$$F_{0}/F=1+K_{Q}[Q]$$

where *K*_Q_ is the Stern–Volmer constant and [*Q*] is the concentration of the quencher. Samples were excited at 280 nm and spectra were taken between 300 nm and 380 nm.

### Folding and unfolding of PagP detected by CD

Formation of secondary and tertiary structure during incubation of 5 μM PagP initially denatured in 6 M Gdn-HCl with *di*C_12:0_PC LUVs in the presence of 7 M urea was followed by changes in the molar ellipticities at 218 nm and 232 nm, respectively. To measure the kinetics of unfolding, PagP was first allowed to refold in liposomes as described above, after which the denaturant concentration was increased to 10 M urea, whilst maintaining all other experimental conditions the same. Similar unfolding rates were obtained at 218 nm and 232 nm (data not shown). Samples were mixed manually, resulting in a dead time of 30 s. All measurements were acquired with a Jasco J715 instrument using a response time of 16 s at 25 °C or 37 °C in a cuvette with a 1 mm path length. Measurements were taken in 50 mM sodium phosphate buffer (pH 8) and averaged over two measurements to increase the signal-to-noise ratio. Folding and unfolding traces were normalised between the signal of the folded and unfolded protein in 7 M and 10 M urea, respectively, and fitted to a single exponential.

### Fourier transform infrared spectroscopy (FTIR)

Attenuated total reflection FTIR measurements were carried out on a Nicolet 560 FTIR spectrometer equipped with a germanium ATR plate. Approximately 300 μg of PagP in 50 mM sodium phosphate buffer (pH 8), containing 1% cyclofos-7, was dried under a gentle stream of nitrogen gas to form a thin film on the surface. Buffer and protein spectra were recorded at a spectral resolution of 4 cm^−1^ by averaging 1024 scans. Spectra were analysed using OMNIC E.S.P. 5.0 and Galactic Peaksolve™ version 1.05 software and component bands in the amide I absorption band were assigned according to Goormaghtigh *et al*.^56^

### Activity assays

The enzymatic assay for PagP in cyclofos-7 was adapted from an assay commonly used for lipase activity.^57^ The enzymatic activity of PagP, refolded in cyclofos-7, towards the substrate analogue *p*-nitrophenylpalmitate was assayed in a final assay volume of 1 ml using a final substrate concentration of 1 mM. The substrate (20 mM in isopropanol) was mixed with 50 mM sodium phosphate (pH 8), containing 2% (v/v) Triton X-100 and 0.05% cyclofos-7 before addition of the enzyme to a final concentration of 2 μM. The rate of reaction was monitored for 15 min, measuring the increase in absorbance at 410 nm upon generation of *p*-nitrophenol upon hydrolysis of *p*NPP by PagP. Controls included measuring the activity of 2 μM lipase from *Candida cylindracea* (62316-10G; Fluka, Biochemica), which showed the rapid formation of *p*-nitrophenol (with a rate of 0.702(±0.066) nmol s^−1^ μM^−1^), as well as PagP denatured by heating to 100 °C for 10 min in refolding buffer, which showed no significant increase in absorbance at 410 nm (with a rate of 0.004 (±0.001) nmol s^−1^ μM^−1^).

To measure enzyme activity of PagP during refolding in lipid vesicles, *p*NPP was added to the liposome solution before sonication to obtain a dispersion of SUVs and *p*NPP, after which PagP was added in the presence of 7 M urea as described above. Substrate conversion was followed at 410 nm for 20 min.

## Figures and Tables

**Table 1:** Enzyme turnover of *p*-nitrophenylpalmitate measured by the release of *p*-nitrophenol with a final enzyme concentration of 2 μM in cyclofos-7

	Enzymatic turnover (nmol min^−1^ μM^−1^)	*K*_Q_ ×10^−3^ (mM^−1^)
Wild-type	0.068 ± 0.011	1.28 ± 0.01
W17A	0.051 ± 0.020	1.15 ± 0.04
R59L	0.053 ± 0.003	1.11 ± 0.04
W17A/R59L	0.048 ± 0.011	1.11 ± 0.02
Δ(1-19)	0.058 ± 0.004	1.10 ± 0.09
*N*-Acetyl tryptophanamide		3.51 ± 0.01

Stern–Volmer constants (K_Q_) derived from KI quenching experiments with 0.5 μM protein in *di*C_12:0_PC LUVs are also shown. All experiments were performed in 50 mM sodium phosphate buffer (pH 8) at 25 °C.

**Table 2:** Component band assignments for wild-type PagP and PagP variants obtained using FTIR

	β-Sheet (%)	α-Helix (%)	Turns (%)	Random (%)
Wild-type	45	20	24	11
W17A	50	14	28	8
R59L	58	17	14	11
W17A/R59L	53	15	24	8
Δ(1-19)	64	11	17	8

All experiments were performed in 50 mM sodium phosphate buffer (pH 8) at 25 °C in cyclofos-7.
